# Description of a New Clade within Subtype 1 of *Betaarterivirus suid 1* Causing Severe Outbreaks in Spain

**DOI:** 10.1128/mra.00304-22

**Published:** 2022-06-02

**Authors:** Gerard E. Martín-Valls, Martí Cortey, Alberto Allepuz, Francesc Illas, Montse Tello, Enric Mateu

**Affiliations:** a Departament de Sanitat i Anatomia Animals, Facultat de Veterinària, Universitat Autònoma de Barcelona, Cerdanyola del Vallès, Spain; b Grup Batallé, Riudarenes, Spain; DOE Joint Genome Institute

## Abstract

This report describes 28 genome sequences from a new clade within subtype 1 of *Betaarterivirus suid 1*, formerly known as porcine reproductive and respiratory syndrome virus 1. All share a potential recombinant pattern, with a highly pathogenic Italian strain as the putative major parental sequence and three other possible parents.

## ANNOUNCEMENT

Currently, the International Committee on Taxonomy of Viruses (ICTV) designates porcine respiratory and reproductive syndrome virus 1 (PRRSV1) as *Betaarterivirus suid 1*, within the *Variarterivirinae* subfamily (1). PRRSV1 is one of the most important pathogens of pigs and causes serious economic losses (2). Within PRRSV1, at least 3 subtypes are recognized. Subtype 1 is the most widely distributed (3), while subtypes 2 and 3 were described in Western Siberia and Eastern Europe, respectively (4). Within each subtype, the genetic diversity is considerable. For PRRSV1, recombinant strains have been repeatedly reported in European countries, occasionally involving different modified live virus (MLV) vaccines (5).

Within the scope of a series of longitudinal studies aiming to evaluate the diversity of PRRSV1 quasispecies and the utility of genome segments as epidemiological markers, serum samples were collected between February and November 2021 from sows and piglets on 11 breeding farms located in Catalonia (northeast Spain), owned by different companies. All farms were suffering severe PRRS outbreaks characterized by high abortion rates and mortality in the sows and increased mortality in the weaners and growers (>20%). Briefly, PRRSV1 was isolated from individual serum samples after a single passage in porcine alveolar macrophages (PAMs). The PAMs were inoculated into Eagle’s minimal essential medium (EMEM), supplemented with penicillin-streptomycin and 7.5% bovine calf serum, and maintained. If a cytopathic effect was observed, supernatant was collected, and total RNA was extracted using TRIzol. Libraries were prepared using the NEBNext RNA library prep kit, without an initial amplification step, and sequenced on the Illumina MiSeq platform (paired-end 251-bp reads). Next-generation sequencing (NGS) reads were trimmed using Trimmomatic v0.36 (6) and mapped against the PRRSV1 prototype strain Lelystad (GenBank accession number NC_043487), using the Burrows-Wheeler Aligner (BWA-MEM algorithm). The SAMtools view (7) was used to remove reads with a mapping quality lower than Q20. LoFreq-Star v2.0 (8) was used to call variants. Bam-readcount v1.0.1 (9) was used to determine the frequency of each base at each position, and this frequency was used to infer a quasispecies population of 100 genomes in FASTA format. Variants with a minimum base-call quality score lower than 20 and coverage less than 5× were discarded. From this quasispecies, a consensus sequence was generated using Consensus Maker (https://www.hiv.lanl.gov/content/sequence/CONSENSUS/consensus.html). All tools were run with default parameters. The whole-genome sequences obtained (Table 1) were compared with other PRRSV1 sequences available in GenBank, as well as in the database of the Veterinary Laboratory for the Diagnosis of Infectious Diseases of the Universitat Autònoma de Barcelona. Phylogenetic analyses were performed using MEGA X. Recombination was detected using RDP5 software and GARD.

**TABLE 1:** Sample and sequencing data for the 28 PRRSV1 genomes described

Sample ID^*a*^	GenBank accession no.	SRA accession no.	No. of reads	Length (bp)	%GC	Coverage (×)
PRRSV1/Pig-wt/CAT-Esp/R1/May-2021	OM893828	SAMN27285123	680,713	14,910	52.8	538
PRRSV1/Pig-wt/CAT-Esp/R2/Feb-2021	OM893829	SAMN27285124	674,411	14,910	52.8	845
PRRSV1/Pig-wt/CAT-Esp/R3/Feb-2021	OM893830	SAMN27285125	829,785	14,910	52.7	3,317
PRRSV1/Pig-wt/CAT-Esp/R4/Jun-2021	OM893831	SAMN27285126	345,101	14,910	52.6	1,147
PRRSV1/Pig-wt/CAT-Esp/R5/Oct-2021	OM893832	SAMN27285127	1,061,085	14,910	52.7	2,691
PRRSV1/Pig-wt/CAT-Esp/R6/Mar-2021	OM893833	SAMN27285128	477,811	14,910	52.8	812
PRRSV1/Pig-wt/CAT-Esp/R7/May-2021	OM893834	SAMN27285129	920,987	14,910	52.7	669
PRRSV1/Pig-wt/CAT-Esp/R8/Jun-2021	OM893835	SAMN27285130	86,290	14,910	52.8	169
PRRSV1/Pig-wt/CAT-Esp/R9/Jul-2021	OM893836	SAMN27285131	878,809	14,910	52.7	1,975
PRRSV1/Pig-wt/CAT-Esp/R10/Apr-2021	OM893837	SAMN27285132	5,653,071	14,910	52.6	2,182
PRRSV1/Pig-wt/CAT-Esp/R11/Apr-2021	OM893838	SAMN27285133	968,821	14,910	52.8	1,328
PRRSV1/Pig-wt/CAT-Esp/R12/May-2021	OM893839	SAMN27285134	561,265	14,910	52.7	609
PRRSV1/Pig-wt/CAT-Esp/R13/Jun-2021	OM893840	SAMN27285135	419,903	14,910	52.7	1,546
PRRSV1/Pig-wt/CAT-Esp/R14/Jul-2021	OM893841	SAMN27285136	933,866	14,910	52.7	1,547
PRRSV1/Pig-wt/CAT-Esp/R15/Nov-2021	OM893842	SAMN27285137	1,292,169	14,910	52.7	2,564
PRRSV1/Pig-wt/CAT-Esp/R16/Nov-2021	OM893843	SAMN27285138	795,071	14,910	52.7	1,855
PRRSV1/Pig-wt/CAT-Esp/R17/Apr-2021	OM893844	SAMN27285139	1,187,971	14,910	52.8	4,345
PRRSV1/Pig-wt/CAT-Esp/R18/Apr-2021	OM893845	SAMN27285140	1,260,111	14,910	52.7	4,168
PRRSV1/Pig-wt/CAT-Esp/R19/May-2021	OM893846	SAMN27285141	440,400	14,910	52.6	353
PRRSV1/Pig-wt/CAT-Esp/R20/May-2021	OM893847	SAMN27285142	763,771	14,910	52.6	1,477
PRRSV1/Pig-wt/CAT-Esp/R21/Jun-2021	OM893848	SAMN27285143	301,518	14,910	52.6	263
PRRSV1/Pig-wt/CAT-Esp/R22/Jul-2021	OM893849	SAMN27285144	790,502	14,910	52.6	1,150
PRRSV1/Pig-wt/CAT-Esp/R23/Mar-2021	OM893850	SAMN27285145	1,008,440	14,910	52.7	2,128
PRRSV1/Pig-wt/CAT-Esp/R24/Mar-2021	OM893851	SAMN27285146	886,568	14,910	52.8	2,154
PRRSV1/Pig-wt/CAT-Esp/R25/Apr-2021	OM893852	SAMN27285147	440,798	14,910	52.8	406
PRRSV1/Pig-wt/CAT-Esp/R26/May-2021	OM893853	SAMN27285148	830,917	14,910	52.7	761
PRRSV1/Pig-wt/CAT-Esp/R27/Jun-2021	OM893854	SAMN27285149	352,456	14,910	52.8	993
PRRSV1/Pig-wt/CAT-Esp/R28/Jul-2021	OM893855	SAMN27285150	716,349	14,910	52.6	839

a Includes host, origin, strain, and sampling date (mo-yr). Pig-wt, XXX; CAT-Esp, Catalonia, Spain.

**FIG 1:**
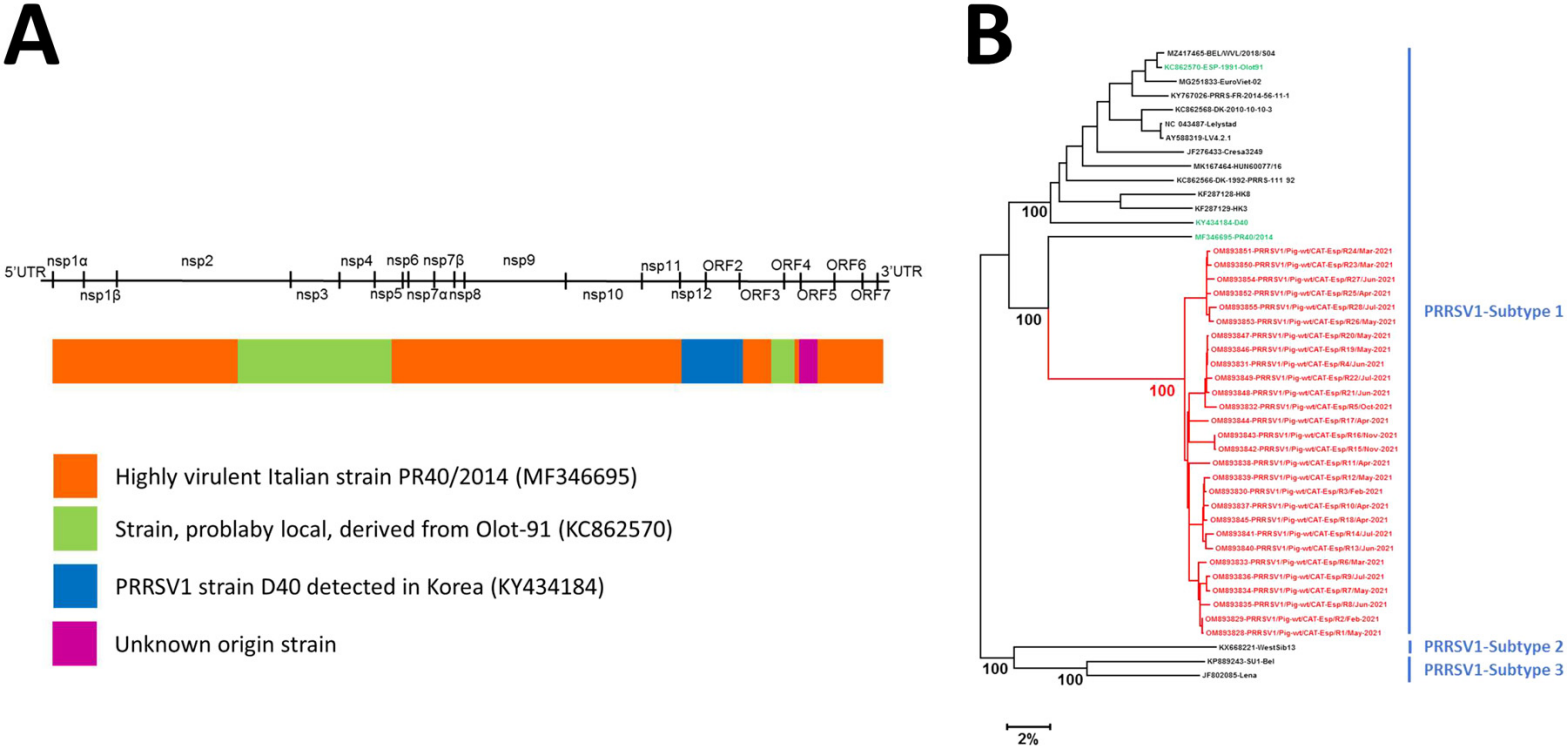
(A) Local similarity analyses among the 28 PRRSV1 genomes described, including the segments associated with every best BLAST hit of genomic subregions, indicated via the color code (identified using RDP and GARD); (B) maximum likelihood phylogenetic tree built up using the whole-genome sequence and the general time-reversible model. The confidence of the main internal branches, based on 100 bootstrap replicates, is indicated. Three PRRSV1 subtypes are marked in blue, the potential recombinant clade described in this report is highlighted in red, and the three putative parental strains identified are indicated in green. ORF, open reading frame; UTR, untranslated region.

Local similarity analyses conducted using RDP and GARD revealed a common pattern among all isolates (Fig. 1A). A local similarity analysis conducted using BLAST identified a highly pathogenic Italian strain (10) as the putative major parental sequence (PR40/2014; GenBank accession number MF346695; 80% of the genome comprised in 5 segments, with 86 to 89% nucleotide identity) and the following three putative minor parental strains: ESP-1991-Olot91 (KC862570; 11% of the genome comprised in 2 segments, with 86% nucleotide identity), D40 (KY434184; 7% of the genome comprised in a single segment, with 86% nucleotide identity), and an unknown strain (2% of the genome comprised in a single segment, with a nucleotide identity lower than 85%).

A maximum likelihood (ML) phylogenetic tree placed all the genomes in a monophyletic branch within PRRSV1 subtype 1 (Fig. 1B).

The strains isolated in these outbreaks, characterized by high virulence, potentially originated from the recombination of multiple subtype 1 strains. Its apparent greater pathogenicity should be evaluated in future studies. Furthermore, the regular detection of recombinant PRRSV strains (5) illustrates the plasticity of this virus, together with the need to use whole-genome sequences as an analytical tool.

### Data availability.

The 28 whole-genome sequences described in this report have been deposited at GenBank under the accession numbers OM893828 to OM893855; the corresponding fastq files have been deposited in the Sequence Read Archive under the BioSample accession numbers SAMN27285123 to SAMN27285150.
